# Bone Environment Influences Irreversible Adhesion of a Methicillin-Susceptible *Staphylococcus aureus* Strain

**DOI:** 10.3389/fmicb.2018.02865

**Published:** 2018-11-27

**Authors:** Fany Reffuveille, Jérôme Josse, Frédéric Velard, Fabien Lamret, Jennifer Varin-Simon, Marie Dubus, Evan F. Haney, Robert E. W. Hancock, Céline Mongaret, Sophie C. Gangloff

**Affiliations:** ^1^EA 4691 Biomaterials and Inflammation in Bone Site (BIOS), SFR Cap Santé (FED 4231), University of Reims-Champagne-Ardenne, Reims, France; ^2^CIRI, INSERM U1111 – CNRS UMR5308 – ENS Lyon, Team “Staphylococcal Pathogenesis”, Lyon 1 University, Lyon, France; ^3^Centre for Microbial Diseases and Immunity Research, Department of Microbiology and Immunology, University of British Columbia, Vancouver, BC, Canada; ^4^Pharmacy Department, University Hospital of Reims, Reims, France

**Keywords:** bone and joint infections, biofilm, antibiofilm peptides, bone environment, bacterial starvation

## Abstract

Prosthesis and joint infections are an important threat in public health, especially due to the development of bacterial biofilms and their high resistance to antimicrobials. Biofilm-associated infections increase mortality and morbidity rates as well as hospitalization costs. Prevention is the best strategy for this serious issue, so there is an urgent need to understand the signals that could induce irreversible bacterial adhesion on a prosthesis. In this context, we investigated the influence of the bone environment on surface adhesion by a methicillin-susceptible *Staphylococcus aureus* strain. Using static and dynamic biofilm models, we tested various bone environment factors and showed that the presence of Mg^2+^, lack of oxygen, and starvation each increased bacterial adhesion. It was observed that human osteoblast-like cell culture supernatants, which contain secreted components that would be found in the bone environment, increased bacterial adhesion capacity by 2-fold (*p* = 0.015) compared to the medium control. Moreover, supernatants from osteoblast-like cells stimulated with TNF-α to mimic inflammatory conditions increased bacterial adhesion by almost 5-fold (*p =* 0.003) without impacting on the overall biomass. Interestingly, the effect of osteoblast-like cell supernatants on bacterial adhesion could be counteracted by the activity of synthetic antibiofilm peptides. Overall, the results of this study demonstrate that factors within the bone environment and products of osteoblast-like cells directly influence *S. aureus* adhesion and could contribute to biofilm initiation on bone and/or prosthetics implants.

## Introduction

Infections on orthopedic implant materials (prosthesis or osteosynthesis support) represent a major threat for public health due to an aging population. The major consequence is an increase in joint infection risks and ultimately replacement of prosthesis. These post-surgical infections occur in 0.5–2% of cases, leading to irreversible sequelae and even death (Grammatico-Guillon et al., 2012). Many mechanisms explain the increased risk of infection due to the presence of a foreign body. Mainly, bacteria are able to adhere to implant surfaces and form biofilms, which are difficult to remove (Ribeiro et al., 2012). Biofilms, a bacterial community forming a multicellular structure, represent an underappreciated growth state and possess a strong capacity to resist very high concentrations of antimicrobial compounds (Høiby et al., 2011; de la Fuente-Núñez et al., 2013b). This resistance is accentuated by the inability of antibiotic treatment to successfully treat bone infections due to pharmacokinetic issues in that the penetration of antibiotics depends on many factors including pharmacological characteristics, the degree of vascularization, access through soft tissues, and the presence of foreign bodies (Lima et al., 2014). For these reasons, the minimal bacterial inoculum required to cause infection on orthopedic materials is lower than in other kinds of infections. Chronic infections associated with biofilms are much more difficult to diagnose (symptoms appearing a long time post-surgery) and treatment typically involves invasive surgical debridement (Lew and Waldvogel, 2004; Mylona et al., 2009; Spellberg and Lipsky, 2012; Jacqueline and Caillon, 2014), which can lead to prolonged and expensive hospitalization. *Staphylococcus aureus* is responsible for 30–80% of bone and joint infections, and this species has been isolated in 12–25% of orthopedic material infections (Lipsky et al., 2007; Grammatico-Guillon et al., 2012; Römling and Balsalobre, 2012; Bhattacharya et al., 2015; Kremers et al., 2015; McCarthy et al., 2015; Edmiston et al., 2016). The presence of *S. aureus* as a commensal in 20% of the human population (up to 60% intermittently) could explain the prevalence of this species in biofilm associated-infections (Kluytmans and Wertheim, 2005; Wertheim et al., 2005). Moreover, *S. aureus* has a strong capacity to adhere to surfaces due to its production of several adhesion molecules, which allow for its attachment to different human matrix proteins (fibronectin, fibrinogen, etc.) and to indwelling medical devices (Otto, 2013). *S. aureus* produces a structure surrounding the cells within the biofilm consisting of a variety of molecules including: accumulation-associated protein, extracellular matrix binding protein, protein A, biofilm-associated surface protein, amyloid proteins (Corrigan et al., 2007; Taglialegna et al., 2016), extracellular DNA (eDNA) which appears through cell lysis (Rice et al., 2007) and exopolysaccharides (EPS). In staphylococci, the major EPS components within the extracellular matrix consist of the polysaccharide intercellular adhesin and poly-*N*-acetylglucosamine (Mack et al., 1996).

There is an urgent need to develop strategies to prevent infections in orthopedic implant patients and avoid biofilm initiation. Unfortunately, signal mechanisms that induce the bacterial biofilm program to switch on and promote bacterial adhesion on an implant or bone surface remain poorly understood. One hypothesis is that bacterial biofilm formation is an environmental stress response. Indeed, the Agr system responsible for quorum-sensing in *S. aureus* is known to be involved as an inhibitor in biofilm formation and its expression depends on environmental conditions (Yarwood et al., 2004). In *S. aureus*, various studies have evaluated the influence of starvation, mineral ions, pH, nitric oxide, dioxygen rate and presence of antibiotics on biofilm development (Regassa and Betley, 1992; Regassa et al., 1992; Cramton et al., 2001; Lim et al., 2004; Fluckiger et al., 2005; de Nys et al., 2006; Schlag et al., 2007; Kuehl et al., 2009; Boles et al., 2010; Nguyen et al., 2011; de la Fuente-Núñez et al., 2013a, 2014; Formosa-Dague et al., 2016). The role of environmental changes in biofilm formation makes sense, since biofilms provide protection for all embedded bacteria against those stresses. Bone is a specific environment with organic components (bone cells and extracellular matrix composed of 80% type I collagen) and it is the principal reservoir of mineral ions (calcium, phosphates, carbonates and magnesium) (Green, 1994). In this context, *S. aureus* could encounter many stresses including lack of oxygen (Xu et al., 2016), excess or lack of specific nutrients, and contact with bone cell products and/or signals (e.g., growth factors, cytokines, hormones). In the current study, we evaluated the impact of the bone microenvironment [containing mineral ions, Ca^2+^and Mg^2+^, and deficient in oxygen and nutrients] as well as the effect of bone cells on an MSSA (Methicillin-Susceptible *S. aureus*) strain, in order to understand the possible inter-species communication between the bacteria and the host. Deciphering which factors contribute to biofilm adherence and maturation in the context of bone tissue is necessary for the creation of an *in vitro* bone-biofilm model that reflects the *in vivo* context of *S. aureus* bone infections.

## Materials and Methods

### Bacterial Strains and Culture Media

*Staphylococcus aureus* CIP 53.154 (sensitivity test organism Quality control strain for European Pharmacopeia) also named ATCC9144 or NCTC 6571 was first isolated in Oxford, United Kingdom in 1944 (Heatley, 1944) and possess the “Set1 gene cluster.” This strain is methicillin sensitive whereas two mutations are known in *pbp2* gene (Fuller et al., 2005) and biofilm former (Diaz De Rienzo et al., 2016). We confirmed that *S. aureus* CIP 53.154 produces exopolysaccharides, proteins and eDNA, components of the biofilm matrix by fluorescent staining according to Trivedi et al. (2016) and by enzymatic digestion of the biofilm (Supplementary Figure S1) (Asai et al., 2015). Briefly, after a 24-h incubation of *S. aureus* culture in a 48-well plate, wells are rinsed with sterile water to eliminate planktonic bacteria. A lysis buffer (100 μL of 20 mM Tris-HCl pH7.5, 100 mM NaCl, 1 mM CaCl2) with or without (control) enzymes (100 μg/mL DNAse I or Proteinase K from sigma) was applied for 90 min at 37°C. After washing the wells to remove lysis buffer and lysed matrix, crystal violet staining was applied to detect the remaining matrix. The values of the wells with lysis buffer alone represent the total bacterial matrix formed. The values of the enzyme wells correspond to the percentage of remaining matrix after lysis. In order to determine the percentage of matrix composed of DNA or proteins, we did the following calculation: 100 – [(remaining matrix (after DNAse I or proteinase K digestion) × 100)/(total matrix)]. *S. aureus* strain was cultivated overnight in nutrient medium. A minimal medium (MM) [62 mM potassium phosphate buffer, pH 7.0, 7 mM (NH_4_)_2_SO_4_, 2 mM MgSO_4_, 10 μM FeSO_4_] containing 0.4% (w/v) glucose and 0.1% (w/v) casamino acids, was used in all biofilm models. This minimal medium was modified according to the conditions tested. The influence of calcium (Ca^2+^) was studied by adding CaCl_2_ at serum concentration (1.2 mM) (Kratz et al., 2004), and annotated as 1x Ca^2+^ and 2x Ca^2+^, equivalent to and 2-fold higher than serum level respectively. Mg^2+^ concentrations were modified by changing the amounts of added MgSO_4_. The absence of glucose, casamino acids or iron were tested by not adding them to the minimal medium preparation.

### Static Biofilm Models

*Crystal Violet staining (CV model)*: As previously described, biofilm biomass was evaluated by crystal violet staining (Reffuveille et al., 2014). Briefly, an overnight culture of *S. aureus* was diluted 1/100 in MM and 500 μL was distributed in each well of a 48-well microtiter plate. After 24 h incubation, the planktonic growth was evaluated by measuring the absorbance at 600 nm (A_600_; results are expressed with the subtraction of the blank: medium without bacteria). The plates were gently washed 3 times and 500 μL of 0.2% of crystal violet was applied for 20 min. After washing, 500 μL of 95% ethanol was added to each well. The absorbance at 595 nm was measured to quantify the amount of biofilm (results are expressed with the subtraction of the blank: medium without bacteria). Pictures of crystal violet staining are shown in Supplementary Figure S2. *Counting model*: The quantity of live adhered bacteria was evaluated following bacteria detachment by ultrasound. As before, a 1/100 diluted overnight culture was distributed in 24-well plates, except in this case a plastic lamella (Thermanox^TM^, Nunc, Denmark) was present at the bottom of the well. After 24h of incubation, the lamella was washed and transferred to a Falcon tube containing 2 mL of minimal media. Bacteria were then detached by exposing the sample to 5 min of ultrasound (40 kHz). A volume of 100 μL from serial dilutions was plated on nutritient agar plates to determine the quantity of attached bacteria. *Anaerobic conditions*: Experiments under anaerobic conditions were performed using the GenBox system (Biomérieux, France). Each experiment was performed at least 3 and up to 9 times, each in triplicate. *Fluorescent staining:* Static biofilms were stained using the LIVE/DEAD BacLight Bacterial Viability kit (Molecular Probes, Eugene, OR, United States), with a ratio of SYTO-9 (green fluorescence, live cells) to propidium iodide (PI) (red fluorescence, dead cells) of 1:5, or with SYTO-9 alone prior to microscopy experiments. Image acquisitions were performed on an Axiovert 200M inverted microscope using a 40x objective and the dedicated Axiovision v 3.2.6 software (Carl Zeiss, Germany). Images acquired on the bottom of the well where biofilms formed were representative of all samples. Equal acquisition times were set for both the SYTO-9 and PI channels in any condition of all experiments. Surface quantification of live and dead bacteria was determined using ImageJ software (v1.50i, National Institutes of Health, Bethesda, MD, United States).

### Scanning Electron Microscopy (SEM)

After incubation, biofilms formed on Thermanox^TM^ lamella were washed 2-times in PBS, then fixed in 2.5% (w/v) glutaraldehyde (Sigma-Aldrich) at room temperature for 1 h. After 2 distilled water rinses, cells were dehydrated in graded ethanol solutions (50, 70, 90, and 100% 2-times) for 10 min. Biofilms were finally desiccated in a drop of hexamethyldisilazane (HMDS, Sigma). After air-drying at room temperature, samples were sputtered with a thin gold-palladium film using a JEOL ion sputter JFC 1100 instrument. Biofilms were observed using a Schottky Field Emission Scanning Electron Microscope (JEOL JSM-7900F). Images were obtained at a primary beam energy of 2 kV (SM-EXG65 electron emitter).

### Dynamic Biofilm Model

The biofilms were established as previously described (de la Fuente-Núñez et al., 2014) except that they were grown for 24 h at 37°C in flow chambers with channel dimensions of 1 by 4 by 40 mm. Briefly, the system was assembled and sterilized by pumping through a 0.5% hypochlorite solution and rinsed with sterile water and medium. After an injection of 400 μl of an overnight culture diluted to an OD_600_ of 0.05, chambers were left without flow for 2 h. Then medium was pumped through the system at a constant rate of 2 ml/h for 24 h. Biofilm cells were stained using the LIVE/DEAD BacLight Bacterial Viability kit (Molecular Probes, Eugene, OR, United States), as described above, or with SYTO-9 alone prior to microscopy experiments. Microscopy was performed using a confocal laser scanning microscope (LSM 710 NLO, ZEISS, Germany) and three-dimensional reconstructions were generated using the Imaris software package (Bitplane AG). Biofilm biovolume (μm^3^) was calculated using Imaris software.

### Evaluation of Osteoblast-like Supernatants

The Saos-2 cell line (ATCC^®^HTB-85^TM^) was cultured at 37°C in a 5% CO_2_ humidified atmosphere in Dulbecco’s modified Eagle’s medium (DMEM-Gibco) supplemented with 10% fetal calf serum (Dutscher) and 1% antibiotic solution PenStrep^®^(Gibco) considered as standard medium (SM). Saos-2 cells were grown to 60–80% confluence in SM then rinsed with sterile Dulbecco’s Phosphate Buffered Saline (DPBS, Gibco) to eliminate antibiotics. Saos-2 cells were further incubated with DMEM and 10% fetal calf serum, without antibiotics, supplemented or not with recombinant human Tumor Necrosis Factor α (TNF α) at 20 ng/mL (R&D Systems) in 25 cm^2^ flasks. After 72 h of incubation, collected supernatants of Saos-2 cells were applied to *S. aureus* cultures in minimal media (50% of supernatants and 50% of MM, named SN 50). In each condition, the initial quantity of bacteria was ∼10^6^ CFU/mL. Bacterial adhesion was evaluated after 24 h of contact in the static biofilm models (described above).

### Evaluation of Antibiofilm Peptides Efficiency

Synthetic antibiofilm peptides 1018 (VRLIVAVRIWRR-NH_2_), 1002 (VQRWLIVWRIRK-NH_2_), 3002 (ILVRWIRWRIQW-NH_2_) and DJK-5 (*VQWRAIRVRVIR*-NH_2_) were synthesized by CPC Scientific Inc. (1018, 1002 and DJK-5) or GenScript (3002) using standard Fmoc solid-phase peptide synthesis procedures. All peptides were purified to >95% by reverse phase high-performance liquid chromatography and their identity was confirmed by mass spectrometry. Peptides 1018, 1002, and 3002 all consisted of natural L-amino acids, while DJK-5 was comprised of D-amino acid enantiomers. Peptides were added at a final concentration of 5 μg/mL in MM for the static biofilm assays in the presence or absence of SN.

### Statistical Methods

The statistical significance of the results was assessed using non-parametric analysis with pairwise tests. The exact non-parametric Wilcoxon Mann Whitney test for independent samples was used (StatXact 7.0, Cytel Inc). Stratification was applied when appropriate. We used non-parametric statistics owing to the lack of a normal distribution of the assessed variables. Stratification allowed the impact of technical variability to be taken into account. Differences were considered significant at *p* < 0.05.

## Results

### Influence of Bone Microenvironment on *S. aureus* Biofilm Formation

#### Ca^2+^ Supplementation

**FIGURE 1:**
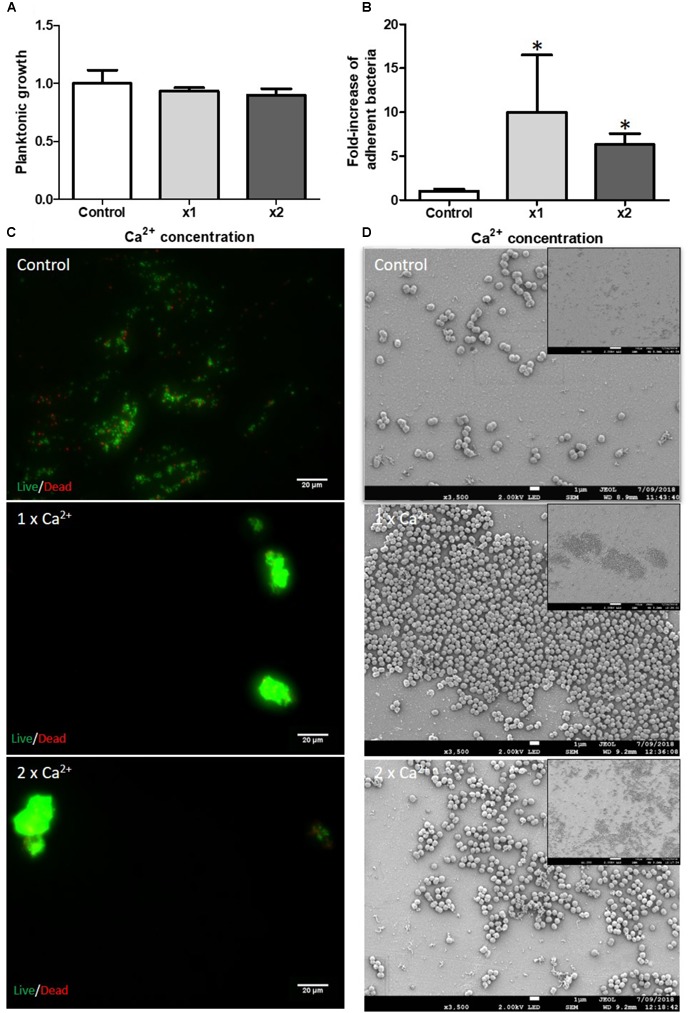
Impact of Ca^2+^ on *S. aureus* biofilm formation. Planktonic growth normalized on control **(A)** and fold-increase of live adhered cells **(B)**. Ca^2+^ × 1 and ×2 serological concentration (=1.2 mM). (*n* = 9). **^∗^**Statistically significantly different from control (*p* < 0.05). Fluorescence microscopy **(C)** with live (green color)/dead (red color) staining. Scale bar = 20 μm. Scanning Electronic Microscopy (SEM) **(D)**. Main panels: scale bar = 1 μm and insert panels showing the homogeneity on a wide field: scale bar = 10 μm.

Bone is mainly composed of calcium phosphate and Ca^2+^ is a mineral ion released in the bone microenvironment. We tested the influence of Ca^2+^ on the initial attachment step of biofilm formation. No impact was observed on planktonic growth (Figure 1A) but there was an increase in numbers of adherent bacteria for the tested Ca^2+^ concentrations (Figure 1B). Quantifying this demonstrated 10-fold and 6-fold more adherent bacteria by counting method in the presence of the serological concentration [x1 = 1.2 mM (Kratz et al., 2004)] and the 2-fold concentration (x2) of Ca^2+^, respectively. Bacteria seemed to aggregate together as observed by fluorescence (Figure 1C) and SEM microscopy (Figure 1D). However, the quantification of the fluorescence signal did not reveal an increase in attached bacteria (data not shown). We supposed that the aggregated bacteria led to fluorescence self-quenching.

#### Mg^2+^ Supplementation

**FIGURE 2:**
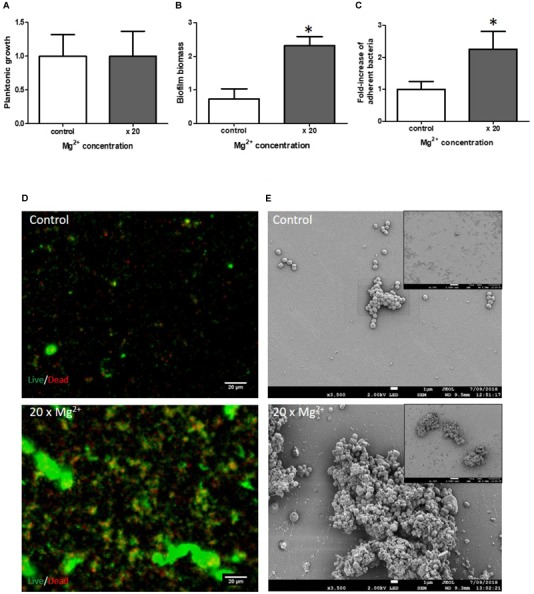
Impact of Mg^2+^ on *S. aureus* biofilm formation. Planktonic growth normalized to control **(A)**; biofilm biomass quantified by crystal violet staining **(B)** and fold-increase of live adhered cells **(C)**. Mg^2+^ × 2 (control) and ×20 serological concentration (=1 mM). (*n* = 9). **^∗^**Statistically significantly different from control (*p* < 0.05). Fluorescence microscopy **(D)** with live (green color)/dead (red color) staining. Scale bar = 20 μm. Scanning Electronic Microscopy (SEM) **(E)**. Main panels: scale bar = 1 μm and insert panels showing the homogeneity on a wide field: scale bar = 10 μm.

Mg^2+^ is a mineral ion present in high abundance in the bone microenvironment but the concentration released from bone is difficult to evaluate. To mimic the release of increasing concentrations of Mg^2+^ by bone reservoir and study the effects of Mg^2+^ on *S. aureus* biofilm growth, we added 20-fold more Mg^2+^ than the concentration found in blood [0.8 – 1.2 mM (Kratz et al., 2004)], a concentration found in rare cases of hypermagnesaemia (Jahnen-Dechent and Ketteler, 2012). Interestingly, 20X serological concentrations (20 mM) had no impact on planktonic growth (Figure 2A). In contrast, maximal biofilm formation was observed at a Mg^2+^ concentration of 20 mM in our two static biofilm models (biomass increased by a factor of 3.18 ± 0.37 fold, *p* = 0.02, while live adhered cells increased by 2.25 ± 0.56 fold, *p* = 0.003) (Figures 2B,C). We quantified the colonized surface area under fluorescent microscopy (Figure 2D), and the ratio of live/dead bacteria in all acquired images thanks to ImageJ software. The increased quantity of bacteria observed in the presence of 20X Mg^2+^ concentration (2.3-fold cf. 2 mM Mg^2+^, *p* = 0.008) was accompanied by a notably higher percentage of dead bacteria (39 ± 8%) (Supplementary Table S2). The SEM analysis did not reveal any impact of Mg^2+^ on bacterial morphology (Figure 2E).

#### Anaerobic Conditions

**FIGURE 3:**
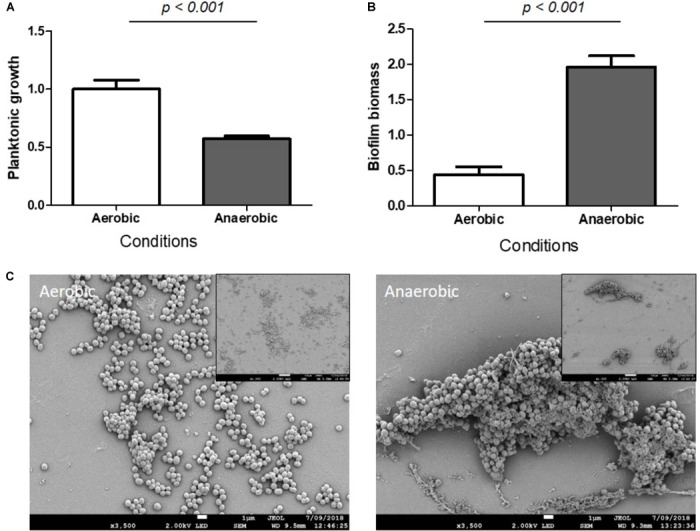
Hypoxia increased *S. aureus* biomass biofilm. Planktonic growth normalized on control **(A)** and biofilm biomass quantified by crystal violet staining **(B)** under aerobic and anaerobic conditions. (*n* = 9). Scanning Electronic Microscopy (SEM) **(C)**. Main panels: scale bar = 1 μm and insert panels showing the homogeneity on a wide field: scale bar = 10 μm.

*Staphylococcus aureus* are facultative aerobes but in the bone microenvironment they suffer from a lack of oxygen (Xu et al., 2016), which is supported by the observation of a decreased planktonic growth under anaerobic conditions (Figure 3A). Anaerobic respiration has been previously shown to enhance *S. aureus* biofilm formation (Cramton et al., 2001; Otto, 2008; Ursic et al., 2008). Our results support these observations as a 4.5-fold increase in biomass was observed in *S. aureus* biofilm grown under anaerobic conditions compared to aerobic conditions, based on crystal violet staining (Figure 3B). SEM microscopy revealed that *S. aureus* produced a more organized structured biofilm under anaerobic conditions (Figure 3C).

#### Starvation

**FIGURE 4:**
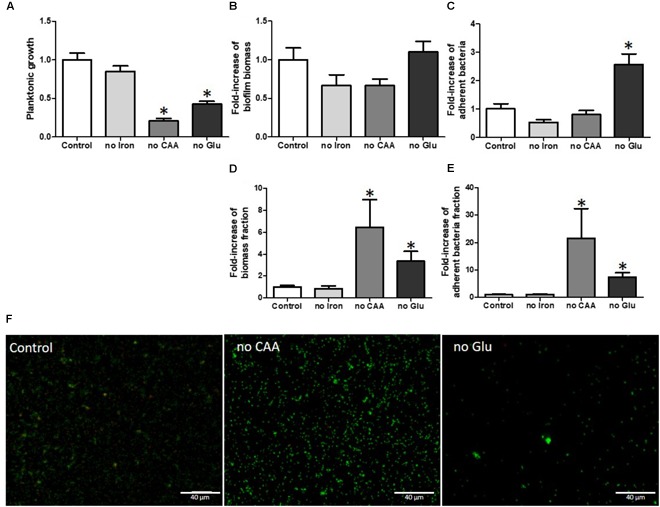
Glucose and amino acid starvation increased *S. aureus* biofilm formation when normalized on planktonic growth. Planktonic growth normalized on control **(A)** fold-increase of biofilm biomass quantified by crystal violet staining **(B)** and fold-increase of live adhered cells **(C)**, fold-increase of biomass fraction on planktonic growth **(D)** and fold-increase of adherent cells fraction on planktonic growth **(E)**. Fluorescence microscopy **(F)** with live (green color)/dead (red color) staining. Scale bar = 40 μm. no iron, without iron; no CAA, without casamino acids; no Glu, without glucose. (*n* = 9). **^∗^**Statistically significantly different from control (*p* < 0.05).

It is well known and obvious that starvation (lack of glucose or amino acids) has a strong impact on the planktonic growth of bacterial cells, findings that were also observed in our experiments (Figure 4A). Iron, an important ion for virulence, was not essential for growth in our model but we could not exclude the presence of residual iron associated with surfaces or carryover with medium (Figure 4A).

With respect to effects on biofilm biomass based on CV staining (Figure 4B), we observed no significant increase of biofilm formation in the absence of any nutrients and only the absence of glucose significantly increased the number of adhered bacteria (Figure 4C). However, the substantial growth defects likely had consequences on biofilm development. Indeed, within a larger bacterial population, adhesion events should be more numerous than in a smaller population. We calculated the ratio of biofilm biomass to planktonic growth, in order to normalize the bacterial content present in the biofilms (Figure 4D). This normalization indicated that media lacking either glucose or casamino acids led to major increases in relative biofilm biomass (4.1-fold and 7.6-fold respectively, *p* = 0.016 and *p* = 0.006). Using the counting method to enumerate live adhered cells we observed the same basic normalized results, namely that the lack of glucose or casamino acids led to relatively increased numbers of live adhered bacteria whereas a deficiency in iron had no effect (Figure 4E).

Using live/dead staining and fluorescence microscopy, no statistical difference was observed in adhesion between CAA-deficient and normal media after quantification of the colonized surface. We noticed a decrease in quantified adhered bacteria in glucose-deficient media, although we did not take into account reduced planktonic growth (Figure 4F). Moreover, the percentage of dead bacteria was reduced in biofilms developed in conditions lacking glucose or casamino acids (CAA) (by 3.24 and 2.13%, respectively), when compared to control medium (12.02%) (Supplementary Table S1).

### Bone Cells

**FIGURE 5:**
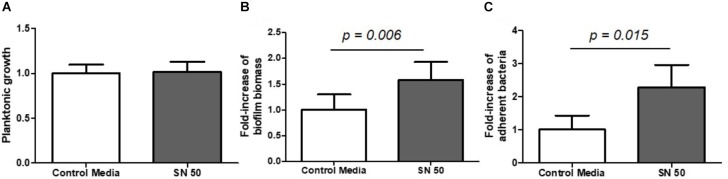
Diluted supernatants of osteoblast culture influenced *S. aureus* biofilm formation. Planktonic growth normalized on control **(A)** biomass biofilm quantified by crystal violet staining **(B)** and fold-increase of live adhered cells **(C)**. Control media = 50% DMEM + 10% FCS and 50% of minimal medium; SN 50 = culture with 50% of osteoblast culture supernatants and 50% of minimal media. (*n* = 9).

Bone is mainly composed of three different types of active cells: osteoblasts, osteoclasts and osteocytes. In this study, we focused on osteoblasts, which are the cells responsible for synthesizing new bone. First, we collected the supernatants of human osteoblast*-*like cell cultures and directly inoculated them with *S. aureus* bacteria in order to study the isolated impact of bone cell products on biofilm initiation. The problem of using supernatants from media in which osteoblasts had already grown was that these cells had consumed most of the available nutrients. This lack of nutrients induced biofilm formation similar to the nutrient-deficient conditions described above (data not shown). We confirmed these findings in cell medium (DMEM, 10% FCS) diluted 1/50 and 1/100 fold into PBS buffer to mimic the osteoblast nutrient-consuming conditions. In this case, we observed an increase of almost 3-fold in biofilm biomass and almost 40-fold in numbers of adherent bacteria in 1/100 diluted medium (Supplementary Figure S3). To avoid this effect, we mixed equivalent volumes of cell culture supernatants and bacterial minimal medium (50% DMEM + 50% MM, termed SN50) to restore some of the nutrients necessary for bacterial growth. Importantly, under these conditions, we did not observe any differences in planktonic growth between supernatants and control conditions (Figure 5A). However SN50 medium increased by almost 2-fold biofilm biomass (Figure 5B, *p* = 0.006) and live adhered cells (Figure 5C, *p* = 0.015).

**FIGURE 6:**
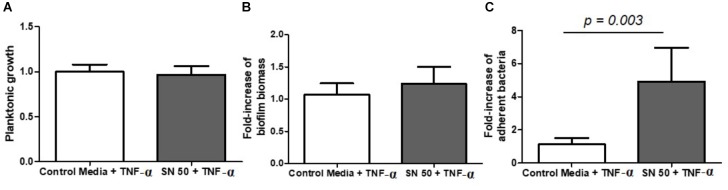
Diluted supernatants of osteoblast culture stimulated with TNF-α influenced *S. aureus* biofilm formation. Planktonic growth normalized on control **(A)** fold-increase of biomass biofilm quantified by crystal violet staining **(B)** and fold-increase of live adhered cells **(C)**. Control medium + TNF-α = 50% DMEM, 10% FCS, 20 ng/ml TNF-α, 50% minimal medium; SN 50 + TNF-α = culture with 50% of osteoblast culture supernatants exposed to 20 ng/ml of TNF-α and 50% of minimal media. (*n* = 9).

Next, we sought to understand the consequence of an inflammatory state on *S. aureus* biofilm growth, so supernatants were used from osteoblast*-*like cells exposed to the pro-inflammatory cytokine TNF-α. Interestingly, supernatants from these stimulated cell cultures appeared to have no impact on planktonic growth (Figure 6A), or on biofilm biomass (Figure 6B), but they substantially increased the number of adherent bacteria by almost 5-fold (Figure 6C).

Indeed, we confirmed this observation by visualizing biofilms grown under the same conditions using a fluorescence microscope (Supplementary Figure S4) demonstrating an increase of 5.1-fold in bacterial adhesion with the addition of SN50 (*p* = 0.021) and 5.3-fold with the addition of TNF-α-stimulated SN50, when compared to control media without any osteoblast supernatant products (*p* < 0.0001) (Supplementary Table S2).

**FIGURE 7:**
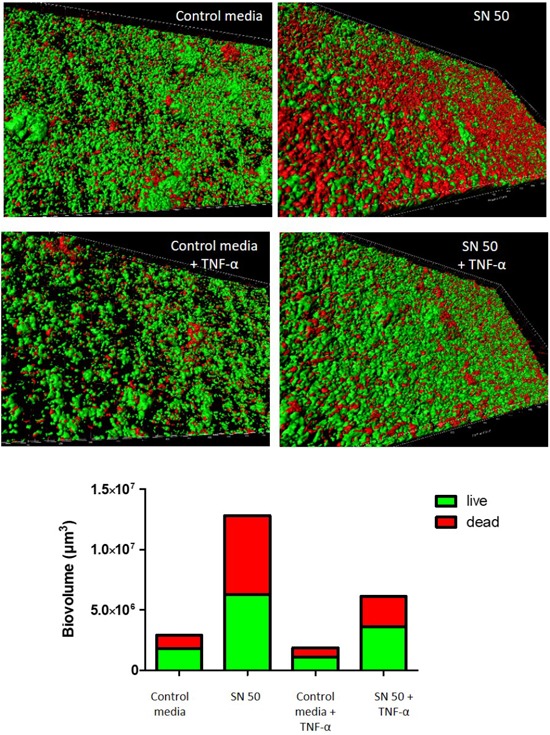
Dynamic model confirmed the impact of osteoblast culture supernatants on *S. aureus* biofilm formation. Impact of osteoblast culture on biofilm was tested into the flow-through medium of the flow cell apparatus for 24 h. Each panel shows 3D reconstruction by Imaris software after acquisition in confocal microscopy with live (green color)/dead (red color) staining. Bottom panel represents the quantitative data calculated Imaris software based on acquired images. Control media, 50% DMEM + 10% FCS and 50% of minimal media; SN 50, culture with 50% of osteoblast culture supernatants and 50% of minimal media; Control media + TNF-α = 50% DMEM, 10% FCS, 20 ng/ml TNF-α, 50% minimal media; SN 50+TNF-α, culture with 50% of osteoblast culture supernatants exposed to 20 ng/ml of TNF-α and 50% of minimal media.

A dynamic biofilm model was used to complement this approach and to model *in vivo* conditions (Figure 7). An increase of biofilm thickness was observed in the presence of osteoblast supernatants with or without TNF-α exposition. We confirmed these findings by quantifying biovolume (Figure 7), revealing a biovolume increase of 4.4-fold and 3.3-fold respectively in the presence of SN50 and TNF-α-treated SN50, when compared to control media. Biofilm structures were similar, but the ratios of live/dead bacteria varied. Indeed, the number of dead bacteria were somewhat higher in presence of osteoblast supernatants without TNF-α (Figure 7).

### Antibiofilm Peptides Prevented the Impact of Osteoblasts on *S. aureus* Biofilm Formation

**FIGURE 8:**
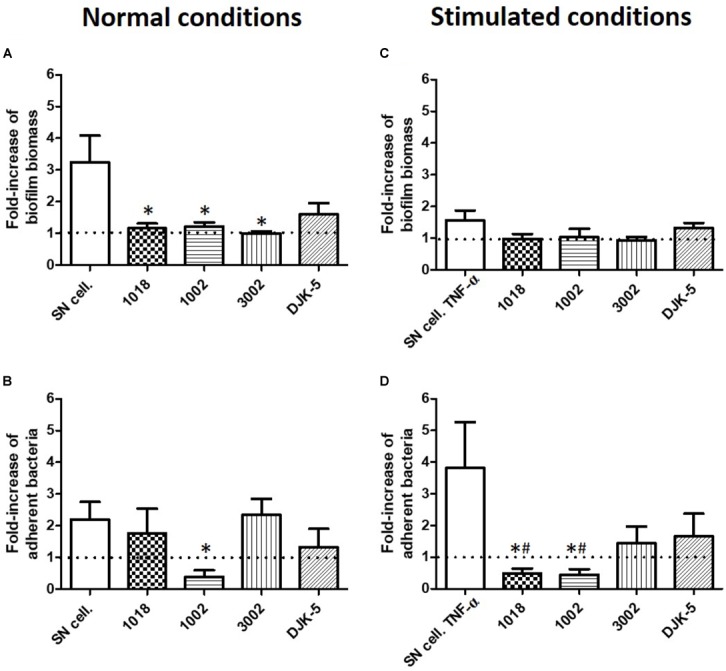
Synthetic peptides (1018, 1002, 3002, and DJK-5, 5 μg/ml) inhibited osteoblast products effect on *S. aureus* biofilm formation. Fold-increase of biofilm biomass quantified by crystal violet staining **(A,C)** and fold-increase of live adhered cells **(B,D)** under normal conditions of osteoblast-like culture **(A,B)** and TNF-α stimulated osteoblast-like culture **(C,D)**. SN cell., increase of biofilm formation in contact of osteoblast supernatants. SN cell. TNF-α, increase of biofilm formation in contact of osteoblast supernatants culture after TNF-α exposition. (*n* = 9). **^∗^**Statistically significantly different from biofilm formed in the presence of SN cell or SN cell. TNF-α (*p* < 0.05). #Statistically significantly different from basal level of biofilm formation. Dotted line = basal level of biofilm formed in the absence of SN cell or SN cell. TNF-α.

To counteract the impact of osteoblast-like cell supernatants on *S. aureus*, we evaluated in our static model the inhibition capacity of four synthetic peptides (1018, 1002, 3002 and DJK-5) previously shown to possess antibiofilm activity against *S. aureus* biofilms (de la Fuente-Núñez et al., 2015; Haney et al., 2015, 2018). The antibiofilm activity of these peptides was assessed at 5 μg/mL which is around the minimal biofilm inhibitory concentrations against *S. aureus* in minimal medium (Figure 8). The biofilm biomass increased in SN50, was reduced by all the peptides (except DJK-5, which was not significant) (Figure 8A). However, only peptide 1002 reduced the quantity of adherent bacteria in the presence of SN50, exhibiting a reduction of 60% (*p* = 0.036) in adhered cells when compared to the medium control (Figure 8B). In biofilms grown in media collected from osteoblast-like supernatants exposed to TNF-α, none of the peptides reduced biomass (Figure 8C), but 1018 and 1002 peptides dramatically reduced the number of adherent bacterial cells (by almost 8-fold, *p* = 0.017 and *p* = 0.029, respectively) (Figure 8D). Moreover, peptides 1018 and 1002 inhibited the number of adherent bacteria by at least 50% compared to medium unstimulated by any osteoblast-like products (below the basal level of biofilm), showing their high potential as antibiofilm molecules (Figure 8D).

## Discussion

In this study, we aimed to mimic the host conditions found within the bone microenvironment *in vitro* to gain insights into the interactions between the bone environment, osteoblasts and *S. aureus* biofilms. In a first approach, we chose to study a MSSA strain used as sensitivity test organism for European Pharmacopoeia. In particular, we focused on the initial bacterial adhesion to a surface, as this would be the first step required for eventual biofilm formation on the surface of bones and implant materials. Moreover, we evaluated bone environment factors that could potentially influence biofilm formation. For instance, the bone microenvironment is primarily composed of Ca^2+^, phosphates and Mg^2+^. Some studies have already shown that calcium and magnesium influence the structure of colony morphology or the slime production of Gram-positive bacteria (Ozerdem Akpolat et al., 2003; Oppenheimer-Shaanan et al., 2016). We used different biofilm models to reinforce these results and evaluated the impact of divalent cations on bacterial multicellularity. We hypothesized that ion concentrations in the bone environment are higher than their serum levels due to active bone modulation or bone resorption especially in the case of an infection and/or inflammation. Ca^2+^ has an important role in many cellular processes and several of the staphylococcal surface adhesins bind the ionized calcium (Arrizubieta et al., 2004). We observed that low concentrations of Ca^2+^ could induce bacterial adhesion (1x and 2x serological concentration). However, a higher concentration (10x serological concentration) had no impact on bacteria attachment (data not shown), revealing the critical role of Ca^2+^ concentrations. As Ca^2+^ levels are highly variable in the bone environment, we conclude that in some specific cases, ionized calcium can influence *S. aureus* biofilm initiation.

Mg^2+^ is another interesting cation as half of the Mg^2+^ reservoir within the body is stored and released from bone (Groisman et al., 2013). We assumed that the Mg^2+^ concentration in the bone environment is higher than the concentration found in plasma. Here, we showed that increasing Mg^2+^ concentration also enhanced biofilm formation at a high concentration (20X plasma concentration) that did not affect bacteria replication. Previous studies have shown that a Mg^2+^ concentration at 3 mM induced the activity of osteoblasts by enhancing gap junction intercellular communication between cells, and influenced bone formation (He et al., 2016). Whereas a higher concentration of Mg^2+^ (at 5 mM) inhibited the differentiation of lineage osteoblasts *in vitro* (Leidi et al., 2011). This highlights the complexity of the magnesium role in bone microenvironment depending on its physiological concentration. The concentration used in this study is strongly exaggerated but it showed that magnesium could play a role in the formation of biofilm especially in case of release of this ion during resorption of bone due to infection or inflammation. We hypothesized that Mg^2+^ might facilitate *S. aureus* adhesion by influencing certain enzymes involved in biofilm formation (Groisman et al., 2013). However, the proportion of dead bacteria in the biofilms formed in the presence of high concentrations of Mg^2+^ was quite high. We therefore speculate that the presence of high Mg^2+^ concentrations might be antagonistic to the survival of bacterial cells within a biofilm even if we did not observe any major differences in SEM microscopy experiments. The cell lysis could also release extracellular DNA that reinforce biofilm structure (Rice et al., 2007).

Environmental stresses are another factor that contribute to biofilm formation and bacteria experience many stresses in the bone microenvironment such as hypoxia and starvation. In this study, we confirmed that anaerobic growth is one stress that induces biofilm formation. The SEM analysis revealed a probable change in the matrix composition, probably due to an abundance of eDNA. Mashruwala et al. (2017) showed an increase of extracellular DNA, probably due to programmed bacterial death, which likely acted as a matrix to confer a robustness to the biofilm. Moreover, glucose or amino acid starvation led to an increase in relative biofilm biomass and live adhered cells, even though the planktonic growth was highly reduced. The high ratio of adherent bacterial numbers compared to the planktonic bacteria numbers indicated that, in a nutrient-deficient microenvironment, most free bacteria preferred to adhere, in agreement with the preliminary studies of Ursic et al. (2008). Nutrient deficient growth conditions appeared to promote biofilm formation. It was previously shown that biofilm production increased in the presence of glucose through increased matrix production (You et al., 2014; Hsu et al., 2015). Here, we observed that the absence of glucose had statistically significant effect on irreversible adhesion. Those results underline the balance between different environmental conditions: the absence of glucose, causing nutrient stress, induces biofilm formation while an excess of glucose reinforces the biofilm matrix development.

Interestingly, iron is one factor known to be essential for *S. aureus* virulence (Hammer and Skaar, 2011). However, we did not observe any impact of iron decrease on biofilm formation but we could not exclude the presence of a very low quantity of ferric ions. Johnson et al. (2005) showed previously that low iron medium could induce biofilm formation, but this effect was strain-dependent, underlining the complexity of iron involvement in biofilm formation. We also observed that biofilms developed under starvation conditions presented a lower proportion of dead bacteria, emphasizing the concept of better survival in biofilms after the induction of a major stress.

The parameters described above represent good mimics of the environment surrounding bones but bone tissue itself is also very active and dynamic: it is continuously remodeled by well-known mechanisms based on cellular signaling communication (Josse et al., 2015). Therefore, in an attempt to mimic these conditions, the supernatants from osteoblast-like cells culture were used to evaluate the impact of osteoblast products on *S. aureus* biofilm adherence and growth. In this model, a growth medium was prepared containing osteoblast products (supernatants of osteoblast*-*like cells culture) and minimal medium at a ratio of 1:1. It was observed that supernatants containing osteoblast-like products enhanced biofilm formation as assessed by increased biomass and greater numbers of adhered bacteria. To mimic the *in vivo* situation encountered during an infection, we also tested the influence of osteoblast-secreted molecules, produced during an inflammatory response (Gallo et al., 2008). Specially, supernatants of osteoblast-like cells exposed to the pro-inflammatory cytokine, TNF-α, were collected and used as the suspension medium to probe *S. aureus* biofilm formation. These growth conditions led to an increase in adherent bacteria that was more pronounced when compared to supernatants obtained under non-inflammatory conditions. We therefore propose that osteoblasts release factors that influence *S. aureus* biofilm formation and that these factors are more prevalent during inflammation. Moreover, it is likely that under inflammatory conditions, bacterial stress is even more evident such that bacteria must rapidly implement survival strategies. Thus, most bacteria in these conditions would strongly adhere to surfaces, enabling the development of a mature biofilm. This might occur even in the absence of major production of matrix (i.e., due to reduced production of eDNA or EPS), which would explain the weak effects on biofilm biomass, but the strong numbers of adherent cells under these conditions. Moreover, the increase of dead bacteria in the biofilm formed in the presence of osteoblast supernatants might be due to the release of bacterial killing molecules from osteoblasts. It would cause some death of adhered bacteria but enable biofilm development regardless, and once bacteria were growing as biofilms they would be far more resistant to external killing by e.g., antibiotics and immune processes. We further propose that extracellular DNA released by such bacteria or those undergoing programmed bacterial death (Bayles, 2014), would stabilize the biofilm structure and lead to an enhanced bacterial resistance. Thus, the study of the matrix composition of biofilms under these conditions warrants further investigation.

Overall, these findings will aid in the construction of an *in vitro* model that more faithfully represents the interactions between the different factors of bone tissue and their potential impact on *S. aureus* biofilm formation. Additionally, deciphering the communications between host cells and bacteria is important for developing appropriate anti-infectious and prevention strategies to limit biofilm-associated infections on bones and orthopedic implants. To that end, we have tested synthetic antibiofilm peptides for their capacity to prevent bacterial adhesion enhanced by osteoblast-released factors in our static (crystal violet and counting) biofilm models. The concentration of peptide used (5 μg/ml) was quite low, had no effect on planktonic bacteria and was previously shown to have no cytotoxic effects (de la Fuente-Núñez et al., 2014, 2015; Haney et al., 2015, 2018). Under both non-inflammatory and inflammatory conditions, peptide 1002 completely prevented the effects of osteoblast supernatants on biofilm biomass and reduced the number of adherent cells to below the threshold of our control. Indeed, peptide 1002 appeared to act as an anti-adhesive molecule in all investigated situations (50% reduction of adhered cells, *p* < 0.05). Peptide 1018 had the same effect on biofilm formation but only when bacteria were co-cultured with supernatants from osteoblast-like cells exposed to TNF-α. These results reveal that 1002 and 1018 inhibited the normal response of bacteria in the presence of osteoblast cell supernatants, possibly through the interference with different bacterial stress signals. In perspective, these results should be confirmed with other *S. aureus* strains such as Methicillin-Resistant *S. aureus* or clinical isolates.

## Conclusion

These findings reflect the possible inter-species interactions and the importance of the specific microenvironment on the growth and proliferation of *S. aureus* biofilm. The bone microenvironment contains many factors that positively influence *S. aureus* biofilm formation. In particular, Mg^2+^ increased *S. aureus* adhesion while the lack of oxygen, starvation and osteoblast signals induced stresses perceived by bacteria, which triggered biofilm formation. Those parameters have to be taken into account in the evaluation of antibiofilm strategies in prosthetic bone and joint infections. One of those strategies could be the use of synthetic peptides to disrupt signals that turn on the bacterial biofilm cellular programming and could serve as possible treatment or prevention strategies for infections associated with orthopedic implants.

## Author Contributions

FR wrote this article, conceived, designed the experiments, and analyzed the data. JJ, FL, JV-S, CM, and MD performed the experiments. FV performed fluorescence and SEM microscopy experiments. EH and RH contributed to peptide experiments. SG analyzed the data in the biofilm experiments. All authors reviewed the manuscript.

## Conflict of Interest Statement

The peptides in this study were invented in part by EH and RH, assigned to their employer the University of British Columbia, filed for patent protection, and licensed to ABT Innovations Inc. The remaining authors declare that the research was conducted in the absence of any commercial or financial relationships that could be construed as a potential conflict of interest.

## Supplementary Material

FIGURE S1 Fluorescent staining and enzymatic digestion to reveal *S. aureus* biofilm matrix components: proteins, eDNA and polysaccharides. *S. aureus* biofilm formation (24 h) was stained with specific fluorochromes: Sypro Ruby (red) for staining proteins, Concanavalin A (blue) for staining α-mannopyranosyl and α-glucopyranosyl residues and Syto9 (green) for live bacteria staining. Fluorescence microscopy with live (green color), Sypro Ruby (red color, merged color with Syto9 is yellow) **(A)** and Concanavalin A (blue color) **(B)**, scale bar = 20 μm. Biofilm (24 h) representation after a 3D reconstruction by Imaris software after acquisition in confocal microscopy with SYPRO Ruby and Syto9 staining, scale bar = 10 μm **(C)**. Extracellular matrix formation was also detected by enzymatic digestion of proteins by proteinase K and DNA by DNAse I **(D)**.

FIGURE S2 Crystal violet staining pictures of *S. aureus* biofilm formation on plastic surfaces. Ctrl, control (no bacteria); MM, Minimal Medium; no glu, MM without glucose; no CAA, MM without casaminoacids; Mg × 20, MM containing × 20 serological concentration of magnesium (=1 mM).

FIGURE S3 Diluted cell medium increased *S. aureus* biofilm formation. **(A)** Planktonic growth normalized to control **(B)** biofilm biomass quantified by crystal violet staining and **(C)** fold-increase of adhesion in the live adhered cells model. *n* = 9.

FIGURE S4 Fluorescence microscopy confirmed the impact of osteoblast culture supernatants on *S. aureus* biofilm formation. Fluorescence microscopy with live (green color)/dead (red color) staining. Control medium, 50% DMEM + 10% FCS plus 50% of minimal media; SN 50, culture with 50% of osteoblast culture supernatants plus 50% of minimal media; Control media + TNF-α, 50% DMEM, 10% FCS, 20 ng/ml TNF-α, 50% minimal media; SN 50 + TNF-α, culture with 50% of osteoblast culture supernatants exposed to 20 ng/ml of TNF-α plus 50% of minimal media.
