# Supplementary figures and images for: Bone Environment Influences Irreversible Adhesion of a Methicillin-Susceptible Staphylococcus aureus Strain
Source: Front Microbiol. 2018 Nov 27;9:2865. doi: 10.3389/fmicb.2018.02865 (PMC6277558; doi:10.3389/fmicb.2018.02865)

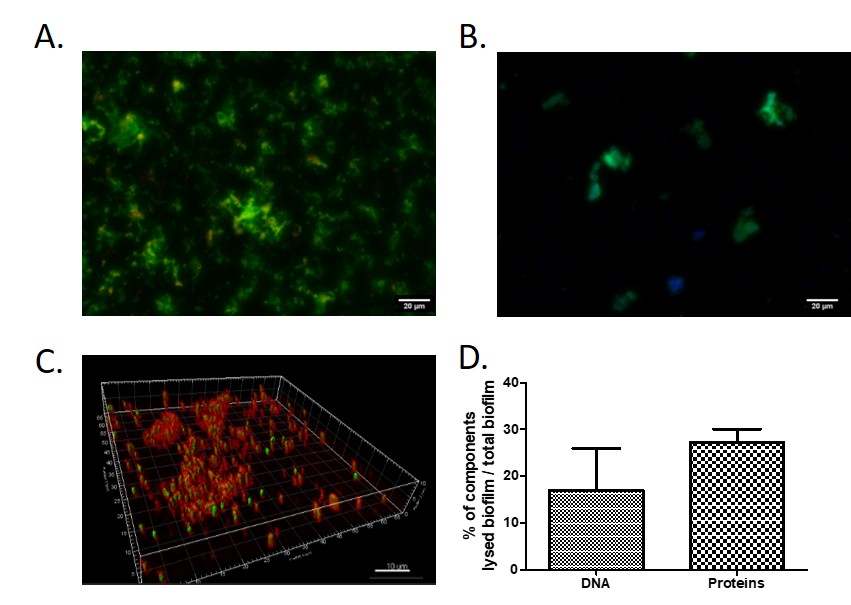

Supplement: FIGURE S1 — Fluorescent staining and enzymatic digestion to reveal S. aureus biofilm matrix components: proteins, eDNA and polysaccharides. S. aureus biofilm formation (24 h) was stained with specific fluorochromes: Sypro Ruby (red) for staining proteins, Concanavalin A (blue) for staining α-mannopyranosyl and α-glucopyranosyl residues and Syto9 (green) for live bacteria staining. Fluorescence microscopy with live (green color), Sypro Ruby (red color, merged color with Syto9 is yellow) (A) and Concanavalin A (blue color) (B), scale bar = 20 μm. Biofilm (24 h) representation after a 3D reconstruction by Imaris software after acquisition in confocal microscopy with SYPRO Ruby and Syto9 staining, scale bar = 10 μm (C). Extracellular matrix formation was also detected by enzymatic digestion of proteins by proteinase K and DNA by DNAse I (D). [file Image_1.JPEG]

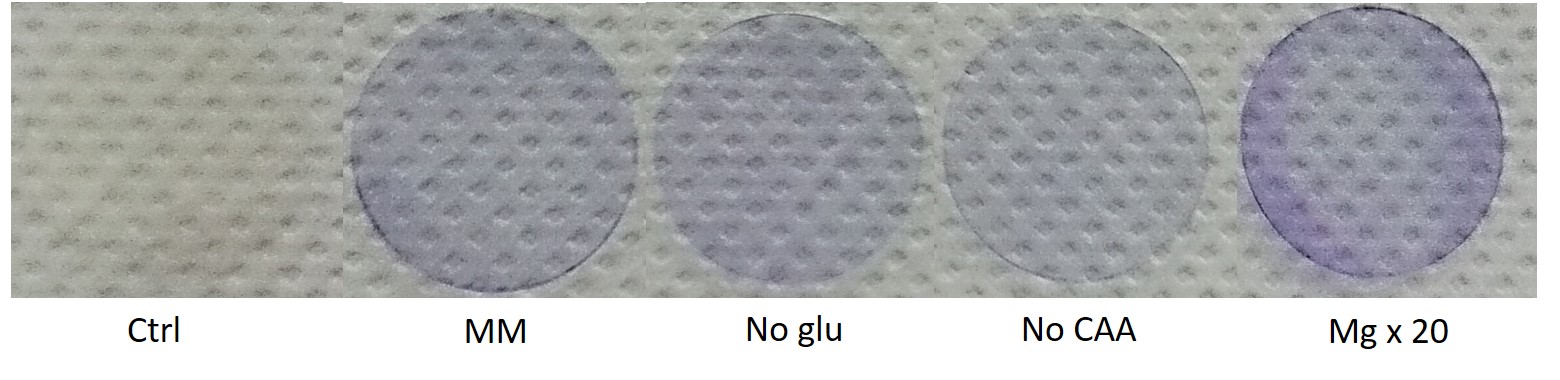

Supplement: FIGURE S2 — Crystal violet staining pictures of S. aureus biofilm formation on plastic surfaces. Ctrl, control (no bacteria); MM, Minimal Medium; no glu, MM without glucose; no CAA, MM without casaminoacids; Mg × 20, MM containing × 20 serological concentration of magnesium (=1 mM). [file Image_2.JPEG]

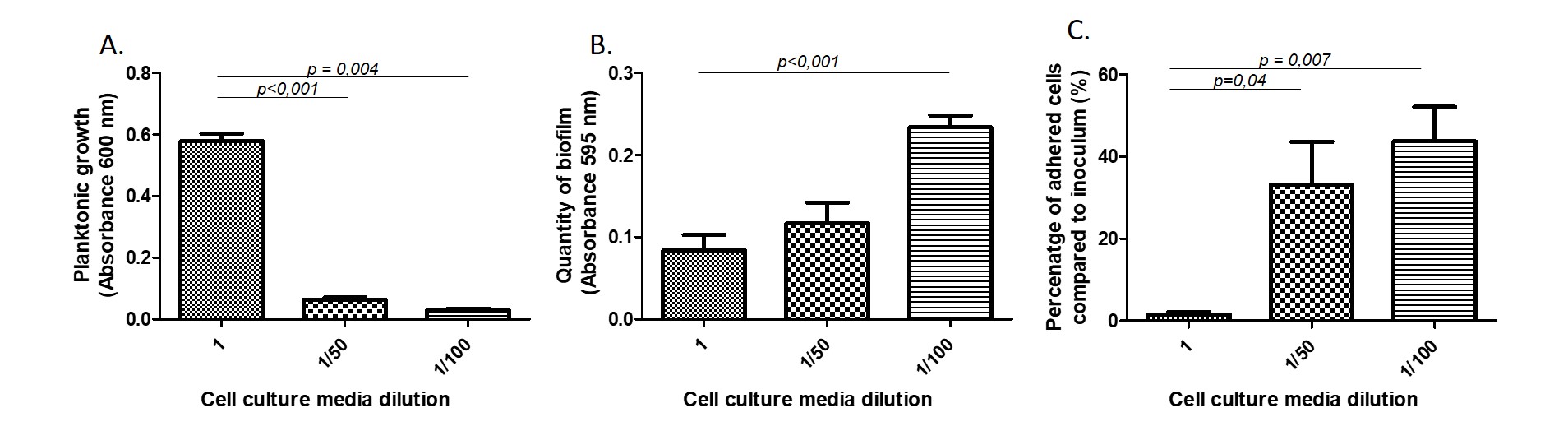

Supplement: FIGURE S3 — Diluted cell medium increased S. aureus biofilm formation. (A) Planktonic growth normalized to control (B) biofilm biomass quantified by crystal violet staining and (C) fold-increase of adhesion in the live adhered cells model. n = 9. [file Image_3.JPEG]

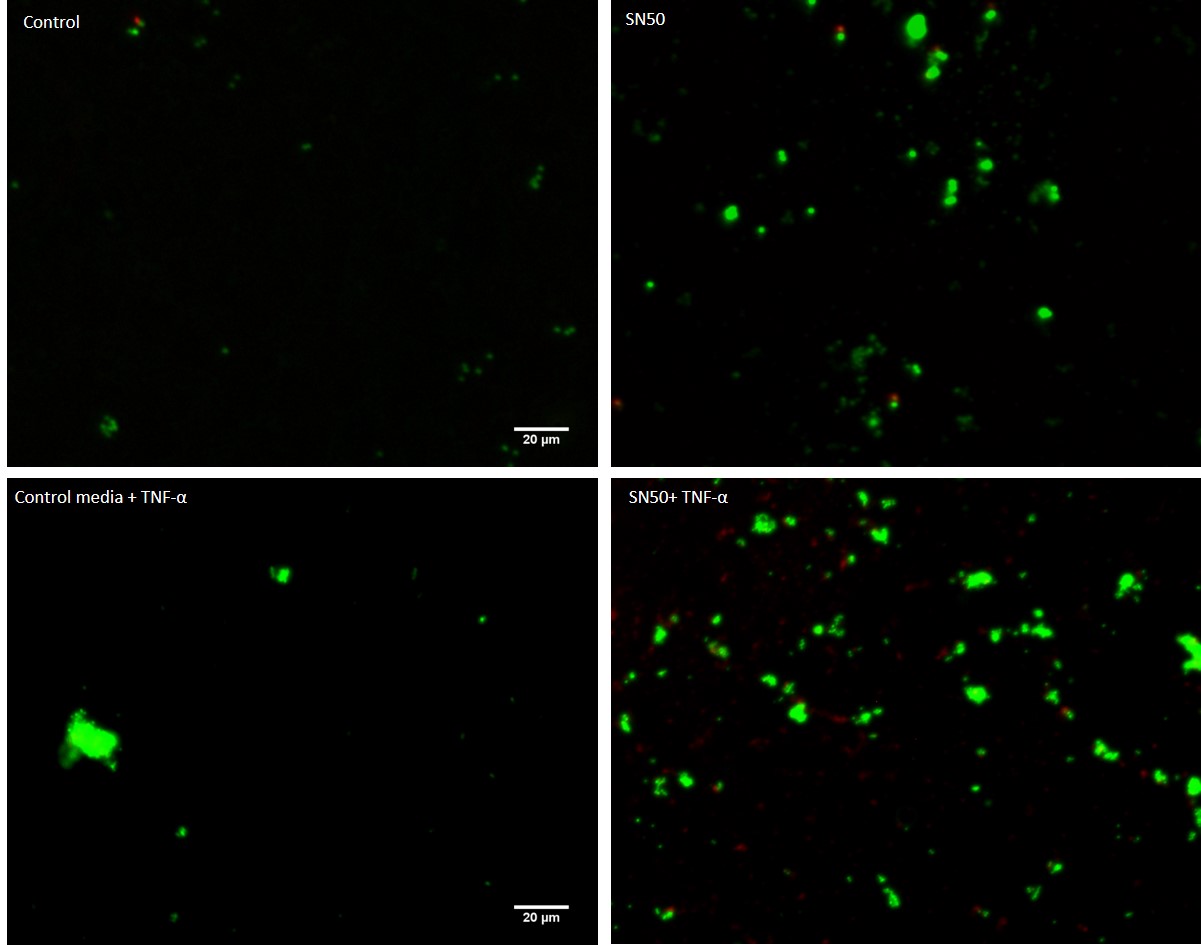

Supplement: FIGURE S4 — Fluorescence microscopy confirmed the impact of osteoblast culture supernatants on S. aureus biofilm formation. Fluorescence microscopy with live (green color)/dead (red color) staining. Control medium, 50% DMEM + 10% FCS plus 50% of minimal media; SN 50, culture with 50% of osteoblast culture supernatants plus 50% of minimal media; Control media + TNF-α, 50% DMEM, 10% FCS, 20 ng/ml TNF-α, 50% minimal media; SN 50 + TNF-α, culture with 50% of osteoblast culture supernatants exposed to 20 ng/ml of TNF-α plus 50% of minimal media. [file Image_4.JPEG]
