# Supplementary material for: Bone Environment Influences Irreversible Adhesion of a Methicillin-Susceptible Staphylococcus aureus Strain
Source: Front Microbiol. 2018 Nov 27;9:2865. doi: 10.3389/fmicb.2018.02865 (PMC6277558; doi:10.3389/fmicb.2018.02865)
Supplement: Supplementary file 5 [file Table_1.DOC]

**Table S1. Nutrient impacts on biofilm formation**: quantification of live/dead surface areas on images acquired thanks to fluorescent microscopy (Image J software). Values with different letters are statistically significantly different from the control (minimal medium).

| Parameter | Minimal Medium | Mgx10 | no CAA | no Glu |
| --- | --- | --- | --- | --- |
| Live cells (% of surface area) | 18.9±6.4 ^a^ | 41.6±9.2^b^ | 11.1±3.8 | 2.99±0.5 |
| Dead cells (% of surface area) | 2.6±0.7 ^a^ | 26.1±6.0^b^ | 0.24±0.1 | 0.1±0.04 |
| Number of Live cells (%) | 87.9±29.8^c^ | 61.4±13.6^d^ | 97.9±33.5^e^ | 96.8±16.2^f^ |
| Number of Dead cells(%) | 12.1±3.3^c^ | 38.6±8.9^d^ | 2.1±0.9^e^ | 3.2±1.3^f^ |
