# Supplementary material for: Bone Environment Influences Irreversible Adhesion of a Methicillin-Susceptible Staphylococcus aureus Strain
Source: Front Microbiol. 2018 Nov 27;9:2865. doi: 10.3389/fmicb.2018.02865 (PMC6277558; doi:10.3389/fmicb.2018.02865)
Supplement: Supplementary file 6 [file Table_2.DOC]

**Table S2.** **Osteoblast-like cells supernatant impacted on biofilm formation**: quantification of live/dead surface areas on images acquired by fluorescence microscopy (Image J software). Values with the letter “a” are statistically significantly different from the control media with or without TNF-α.

| Parameter assessed | % of Surface Area | | | |
| --- | --- | --- | --- | --- |
|  | Control media | SN 50 | Control media + TNF-α | SN 50 + TNF-α |
| Live | 0.54±0.08 | 2.74±0.82^a^ | 0.59±0.11 | 3.11±0.7^a^ |
| Dead | 0.02±0.01 | 0.17±0.09^a^ | 0.03±0.01 | 0.3±0.1^a^ |
